# Identification of masses in digital mammogram using gray level co-occurrence matrices

**DOI:** 10.2349/biij.5.3.e17

**Published:** 2009-07-01

**Authors:** A Mohd. Khuzi, R Besar, WMD Wan Zaki, NN Ahmad

**Affiliations:** 1 Faculty of Engineering, Multimedia University, Cyberjaya, Malaysia; 2 Faculty of Engineering and Technology, Multimedia University, Melaka, Malaysia; 3 Department of Electrical, Electronics and Systems, Faculty of Engineering and Built Environment, Universiti Kebangsaan Malaysia, Selangor, Malaysia

**Keywords:** Breast cancer, Mammogram, Masses, GLCM

## Abstract

Digital mammogram has become the most effective technique for early breast cancer detection modality. Digital mammogram takes an electronic image of the breast and stores it directly in a computer. The aim of this study is to develop an automated system for assisting the analysis of digital mammograms. Computer image processing techniques will be applied to enhance images and this is followed by segmentation of the region of interest (ROI). Subsequently, the textural features will be extracted from the ROI. The texture features will be used to classify the ROIs as either masses or non-masses. In this study normal breast images and breast image with masses used as the standard input to the proposed system are taken from Mammographic Image Analysis Society (MIAS) digital mammogram database. In MIAS database, masses are grouped into either spiculated, circumscribed or ill-defined. Additional information includes location of masses centres and radius of masses. The extraction of the textural features of ROIs is done by using gray level co-occurrence matrices (GLCM) which is constructed at four different directions for each ROI. The results show that the GLCM at 0º, 45º, 90º and 135º with a block size of 8X8 give significant texture information to identify between masses and non-masses tissues. Analysis of GLCM properties i.e. contrast, energy and homogeneity resulted in receiver operating characteristics (ROC) curve area of *Az* = 0.84 for Otsu’s method, 0.82 for thresholding method and *Az* = 0.7 for K-mean clustering. ROC curve area of 0.8-0.9 is rated as good results. The authors’ proposed method contains no complicated algorithm. The detection is based on a decision tree with five criterions to be analysed. This simplicity leads to less computational time. Thus, this approach is suitable for automated real-time breast cancer diagnosis system.

## INTRODUCTION

Breast cancer has become a significant health problem in the world. Early detection is the primary solution for improving breast cancer prognosis. Screening can be done through digital mammogram, ultrasound, magnetic resonance imaging (MRI) or breast biopsy. Ultrasound produces a good contrast image but it does not contain enough detailed information which can be found in digital mammogram. Due to this reason, ultrasound is not approved by the U.S Food and Drug Administration (FDA) as a screening tool for breast cancer [1]. Although MRI is more sensitive than digital mammogram, its results can also lead to false positive diagnosis which then leads to unnecessary additional tests, biopsies and increased patient anxiety. In addition, the American Cancer Society recommends MRI for women with approximately 20-25% or greater lifetime risk of breast cancer, including women with strong family history of breast or ovarian cancer [2]. The benefit of digital mammogram in helping to detect breast cancer early, obviously outweigh the other methods discussed previously. This support the fact that many studies have found that digital mammogram is better at detecting early stage breast cancer [3-14, 16-21].

Although digital mammogram has been proven to be an effective method for detecting breast cancer, interpretation of such mammograms requires skill and experience by a trained radiologist. It is noted that about 10-30% of breast lesions are missed during routine screening [3]. Independent double reading by two radiologists has been shown to improve the sensitivity, but it also increased the cost of the screening process [4]. Thus, computer-aided detection (CAD) can act as a second reader where the final decision will be made by the radiologist.

The combination of CAD scheme and an expert’s knowledge will greatly improve the detection accuracy. CAD system has been developed in mammogram for detection of either mass or micro-calcification (MCC). Detection of masses using digital mammogram is more challenging because masses are usually indistinguishable from the surrounding breast tissues. Normally, masses are hidden or are found in the dense area and are similar to other normal tissues in the breast. Some of the typical shapes of masses are spiculated, circumscribed and ill-defined. Irregular shapes of masses are usually found to be malignant.

Several studies were published on the automatic detection of masses exploring varieties of computational techniques [4, 5-12, 18-21]. Some researchers used multi-view images in CAD system [5, 6]. In particular, Bovis and Singh proposed bilateral subtraction of both mammograms (right and left) in order to find asymmetries in either mammogram [5]. This method, however, leads to a low sensitivity and high false positive rate due to instrinsic breast asymmetry. In their work, classification is based on texture features extracted from the region of interest (ROI). Exploiting information in two views of the same breast taken at some oblique angles, mediolateral oblique (MLO) and craniocaudal (CC), also improved the performance of CAD [6].

Most CAD only uses a single mammogram. He Wan *et al.* carried out pre-processing procedure using exponential transformation and then extracted six features from the suspicious region [7]. The binary decision tree is adopted as classifier due to its conceptual simplicity and computational efficiency. Mohamed and Kadah proposed a three-step system, namely the ROI extraction, features extraction and classification. A set of 88 features are extracted and they found that 78 of those features are capable of discriminating between normal and abnormal breast tissues in digital mammogram with True Positive (TP) rate of 83.3% [8]. Dominguez and Nandi also built a three-step CAD system, but the steps involved were pre-processing, segmentation and elimination of false positive findings. In their pre-processing step, wavelet decomposition and reconstruction, morphological operations and local scaling are used for enhancement of digital mammogram. Then, the segmentation process is performed via conversion to binary images at multiple threshold levels and 18 features are extracted and used for detection with TP rate of 80% [9]. Automatic detection of masses using artificial neural network (ANN) has also been considered by a few researchers [11, 12]. Weidong Xu *et al.* proposed a new algorithm based on ANN for detecting masses automatically [11]. In fatty tissues, iterative thresholding was applied to locate masses and for masses in dense tissues, black hole registration based on discrete wavelet transform (DWT) was used instead. Finally, the segmented suspicious masses were filtrated using 10 selected features via multilayer perceptrons (MLP) classifier, which gave a TP rate of 93.6%. Guodong Zhang et al. used automatic segmentation, 10 selected features for detection of a suspicious area and achieved sensitivity of 83.3% [12].

**Figure 1:**
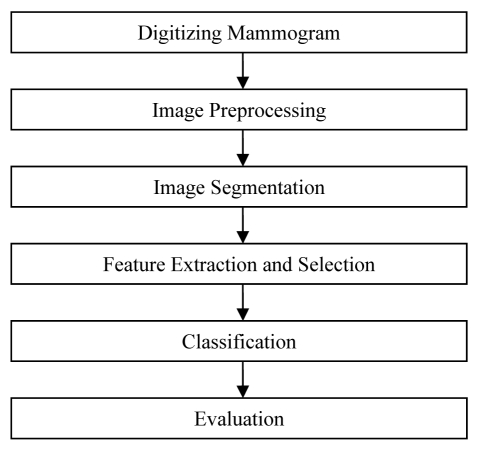
The proposed method of detection for masses in digital mammogram.

Based on the above literature, a better detection rate can be achieved with more features included in the system. However, having more features increase the complexity and time used to analyse the digital mammogram. In this paper, the authors would like to propose a simple CAD system to automatically detect areas that have a high probability of masses in digital mammogram. The detection process is illustrated in Figure 1. This system uses only four features and gives relatively good TP rate as compared to the literature discussed above.

## METHODOLOGY

### Digital mammogram database

The mammogram images used in this experiment are taken from the mini mammography database of MIAS (http://peipa.essex.ac.uk/ipa/pix/mias/). In this database, the original MIAS database are digitized at 50 micron pixel edge and has been reduced to 200 micron pixel edge and clipped or padded so that every image is 1024 X 1024 pixels. All images are held as 8-bit gray level scale images with 256 different gray levels (0-255) and physically in portable gray map (pgm) format. This study solely concerns the detection of masses in mammograms and, therefore, a total of 100 mammograms comprising ill-defined, spiculated, circumscribed and normal case were considered. Ground truth of location and size of masses is available inside the database.

### Pre-processing

Mammograms are medical images that are difficult to interpret, thus a pre-processing phase is needed in order to improve the image quality and make the segmentation results more accurate. The first step involves the removal of artefact and unwanted parts in the background of the mammogram. Then, an enhancement process is applied to the digital mammogram.

**Figure 2:**
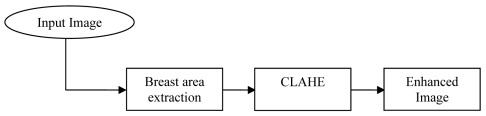
Image pre-processing block diagram.

The contrast limited adaptive histogram equalization (CLAHE) method seeks to reduce the noise produced in homogeneous areas and was originally developed for medical imaging [15]. This method has been used for enhancement to remove the noise in the pre-processing of digital mammogram [16]. CLAHE operates on small regions in the image called tiles rather than the entire image. Each tile’s contrast is enhanced, so that the histogram of the output region approximately matches the uniform distribution or Rayleigh distribution or exponential distribution. Distribution is the desired histogram shape for the image tiles. The neighbouring tiles are then combined using bilinear interpolation to eliminate artificially induced boundaries. The contrast, especially in homogeneous areas, can be limited to avoid amplifying any noise that might be present in the image. The block diagram of pre-processing is shown in Figure 2. The experimental results of enhancement on digital mammogram using CLAHE have been reported in the authors’ previous work [17].

### Segmentation

**Figure 3:**
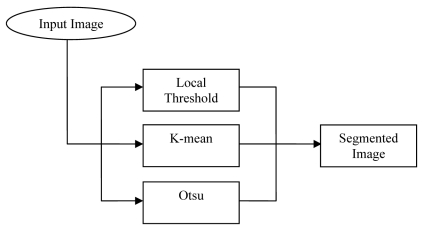
Segmentation block diagram.

In analyzing mammogram image, it is important to distinguish the suspicious region from its surroundings. The methods used to separate the region of interest from the background are usually referred as the segmentation process. The segmentation block diagram is shown in Figure 3. The first method used in this study is the local threshold technique. This technique has been proven to provide an easy and convenient way to perform the segmentation on digital mammogram [13]. The segmentation is determined by a single value known as the intensity threshold value. Then, each pixel in the image is compared with the threshold value. Pixel intensity values higher than the threshold will result in a white spot in the output image. Therefore, experimental work has been conducted and also reported in the authors’ previous work. The results show the detection of ROI that contain masses is 96% [17].

**Figure 4:**
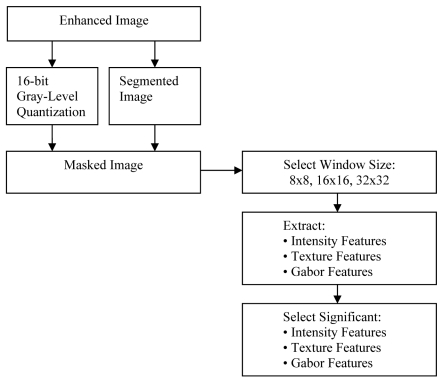
Features extraction and selection block diagram.

For comparison, another two methods of segmentation have been investigated. K-mean clustering is a region clustering method that does not need prior information or start point and is based on an iterative process [13]. This method only requires a stop function, which is the number of clusters, *k*, in the segmented image. The higher the *k* value, the clearer the segmentation but the processing time will increase. An experiment was conducted and the optimum value of *k*, as used in this study, was found to be 4. K-mean segmentation output gives a TP rate of 96%.

The second method for comparison is the Otsu’s method, which has shown a more satisfactory performance in the medical image segmentation. It has been found to perform well compared to other thresholding methods in segmenting the masses in digital mammogram [18]. In this study, the Otsu’s method is able to segment the ROI with a TP rate of 90%.

**Figure 5:**
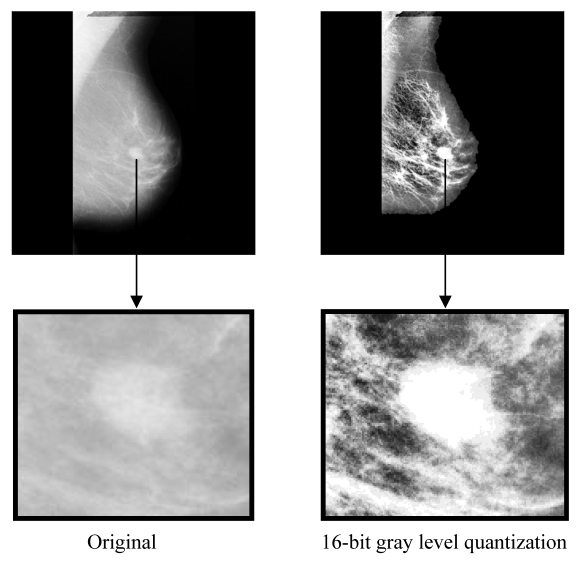
16-bit gray level quantization provides texture information better than original image.

The output of a segmentation process is a binary image. In order to retrieve the texture information, the segmented image is masked with a 16-bit quantization image. Instead of using the original image, a quantized image is used. In the quantized image, the amount of represented intensities is visible to humans. By reducing quantization level to 16 bits, the area with masses can be identified on the mammogram as shown in Figure 5. The masked image is then used as input for the features extraction process.

### Features extraction and selection

Texture features have been proven to be useful in differentiating masses and normal breast tissues [5, 16, 19]. Texture features are able to isolate normal and abnormal lesion with masses and microcalcifications, yielding values of 0.957 and 0.859, respectively, from the area under the curve (ROC) [21]. In the authors’ experimental work, the texture features are extracted using gray level co-occurrence matrices (GLCM). The matrices are constructed at a distance of *d* = 1 and for direction of *θ* given as 0°, 45°, 90° and 135°. A single direction might not give enough and reliable texture information. For this reason, four directions are used to extract the texture information for each masses and non-masses tiles area.

The texture descriptor derived from GLCM are contrast, energy, homogeneity and correlation of gray level values. Table 1 provides the equations for the four features. The contrast measures the amount of local variations present in an image, while energy is the sum of squared elements in GLCM. Energy may also be referred as uniformity or the angular second moment. The homogeneity descriptor refers to the closeness of the distribution of elements in GLCM to the GLCM diagonal. Lastly, correlation will show how correlated a pixel is to its neighbour over the whole image.

**Table 1:** Features of GLCM

**Feature**	**Formula**
**Contrast**	$\sum_{i,j=0}^{N-1}P_{ij}{\left(i-j\right)}^{2}$
**Energy**	$\sum_{i,j=0}^{N-1}{\left(P_{ij}\right)}^{2}$
**Homogeneity**	$\sum_{i,j=0}^{N-1}\frac{P_{ij}}{1+{\left(i-j\right)}^{2}}$
**Correlation**	$\sum_{i,j=0}^{N-1}P_{ij}\frac{\left(i-\mu )(j-\mu \right)}{\sigma^{2}}$

*P_ij_* = Element i, j of the normalized symmetrical GLCM.

*N* = Number of gray levels in the image as specified by number of levels in under quantization on the GLCM.

*μ* = The GLCM mean, calculated as: $\mu =\sum_{i,j=0}^{N-1}iP_{ij}$

*σ*^2^ = The variance of the intensities of all reference pixel in the relationships that contributed to the GLCM, calculated as: $\sigma^{2}=\sum_{i,j=0}^{N-1}P_{ij}{\left(i-\mu \right)}^{2}$

**Figure 6:**
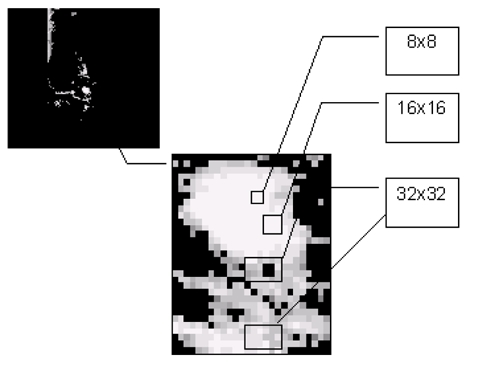
Selection of area for features extraction process.

Based on the authors’ database, the biggest masses area is within a 32x32 window and the smallest is within 8x8 window. Therefore, the authors use window sizes of 8x8, 16x16 and 32x32 for this study. Features extraction and selection block diagram is shown in Figure 5. The processing window or tiles is important because it will determine the ability of the texture descriptor to differentiate between the masses and the normal breast tissues. Note that the selection of area should be done randomly. As illustrated in Figure 6, masses and non-masses areas are captured using 8x8, 16x16 and 32x32 windows.

### Detection of masses

Detection is important in selecting the candidate regions that highly resemble masses in terms of their intensity and statistical texture value. The process is done based on block processing windows or tiles. Therefore, the entire mammogram is divided into tiles area before extraction of features is done to each tile. In this work, detection is implemented in two phases. Phase I would be a preliminary round for detecting any suspicious area with windows of bigger sizes. Thus, the segmented image is divided into tiles with a size of 32x32 and a tile would be categorized as suspicious if its average intensity is more than 200. This threshold value is chosen after extensive investigation on pixel intensity of masses areas. The intensity comparison is applied to each region in the segmented image and regions or tiles that do not fall into this category are rejected.

**Figure 7:**
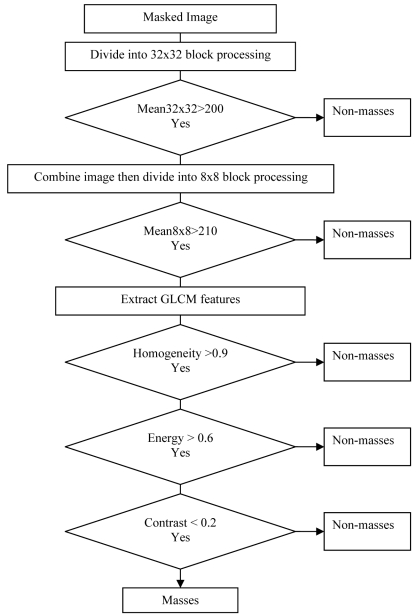
Decision tree for masses detection using GLCM features.

Those regions which are qualified in phase I will be taken as inputs for phase II. Phase II involves a more detailed process. The 32x32 windows are divided into smaller windows with size of 8x8. Then, a tile is considered to be suspicious if its average intensity within 8x8 tiles is more than 210. After that, its texture criteria are evaluated. The tiles are considered as masses if their texture criteria values are within the range of masses texture values. The overlap criterion is used for validation of the proposed method. The flow of detection process is illustrated in Figure 7. The detection is considered true positive (TP) if the region of interest overlaps with the area of groundtruth circle, otherwise the detection is a false positive (FP). Efficiency of detection system is analysed based on four cases, which are false positive (FP), false negative (FN), true positive (TP), and true negative (TN). The definitions of these cases are given in Table 6

**Table 6:** Defination of detection cases

**Cases**	**Meaning**
**TP**	Masses present and result detected masses
**FN**	Masses present and result not detected masses
**TN**	Masses absent and result not detected masses
**FP**	Masses absent and result detected masses.

To evaluate the performance of detection, specificity and sensitivity of detection have been considered. Sensitivity and specificity are terms that show the significance of a test related to the presence or absence of the disease. Equations (2.1) and (2.2) are used to calculate these two parameters, respectively.

(2.1) $$Sensitivity=\frac{TP}{TP+FN}$$

(2.2) $$Specificity=\frac{TN}{TN+FP}$$

In particular, sensitivity indicates the number of subjects who have the disease and are accurately identified by positive test. Thus, it is a measure of the probability of correctly diagnosing a condition. Specificity indicates the number of subjects who do not have the disease, and are accurately identified by negative test. Thus, it is a measure of the probability of correctly distinguishing when the condition is not present in a subject.

Other statistical method known as relative operating characteristic (ROC) curve is also used to analyse the experimental results. ROC curve is a graphical plot of the sensitivity against specificity for a binary classifier system as its discrimination threshold is varied [22]. The ROC can also be represented equivalently by plotting the fraction of true positive rate (TPR) against the fraction of false positive rate (FPR). A ROC curve demonstrates the trade off between sensitivity and specificity in which the closer the curve to the 45° diagonal of the ROC space, the less accurate the test. At the same time, the area under the curve is also a measure of the accuracy. ROC curve in this study is plot using Analyse-it software. An area of 1 represents a perfect test, while an area of 0.5 represents a worthless test.

## RESULTS

In this work, 20 abnormal regions have been used for groundtruth purpose and another 20 normal images were randomly selected for texture extraction. The range of values contrast, homogeneity, energy and correlation of masses and non-masses tissues of 8x8 tile area are shown in Table 2, Table 3, Table 4 and Table 5, respectively.

**Table 2:** Contrast values of masses and non-masses (Tile=8x8)

**W = 8x8**	**Contrast at direction θ**			
	**0°**	**45°**	**90°**	**135°**
**Masses**	0.00-0.07	0.00-0.12	0.00-0.14	0.00-0.16
**Non-masses**	0.27-0.73	0.35-0.82	0.18-0.91	0.39-1.27

**Table 3:** Homogeneity values of masses and non-masses (tile=8x8)

**W = 8x8**	**Homogeneity at direction θ**			
	**0°**	**45°**	**90°**	**135°**
**Masses**	0.96-1.00	0.94-1.00	0.93-1.00	0.92-1.00
**Non-masses**	0.63-0.80	0.61-0.83	0.70-0.91	0.61-0.83

**Table 4:** Energy values of masses and non-masses (Tile=8x8)

**W = 8x8**	**Energy at direction θ**			
	**0°**	**45°**	**90°**	**135°**
**Masses**	0.70-1.00	0.69-1.00	0.67-1.00	0.66-1.00
**Non-masses**	0.16-0.36	0.15-0.36	0.15-0.37	0.14-0.35

**Table 5:** Correlation values of masses and non-masses (tile=8x8)

**W=8x8**	**Correlation at direction θ**			
	**0°**	**45°**	**90°**	**135°**
**Masses**	0.63-0.94	0.00-0.54	0.02-0.61	0.00-0.70
**Non-masses**	(-0.46)-0.82	(-0.55)-0.79	0.14-0.82	(-0.55)-0.74

**Figure 8:**
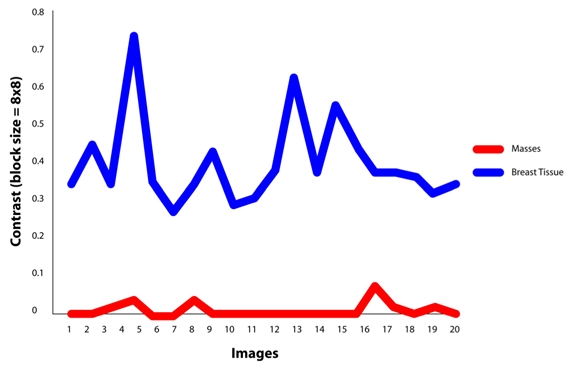
Contrast value at θ=0°using tile 8x8 pixels.

**Figure 9:**
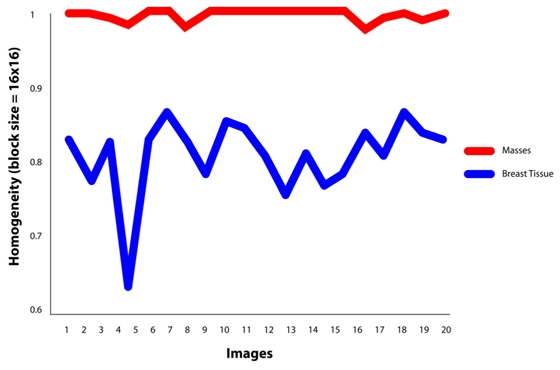
Homogeneity value at θ=0°using tile 8x8 pixels.

**Figure 10:**
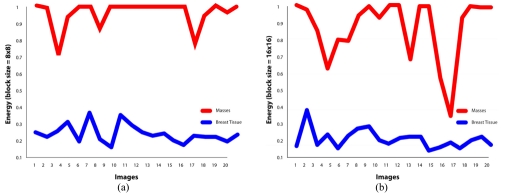
(a) Energy value at θ=0°using tile 8x8 pixels. (b) Energy value at θ=0°using tile 16x16 pixels

**Figure 11:**
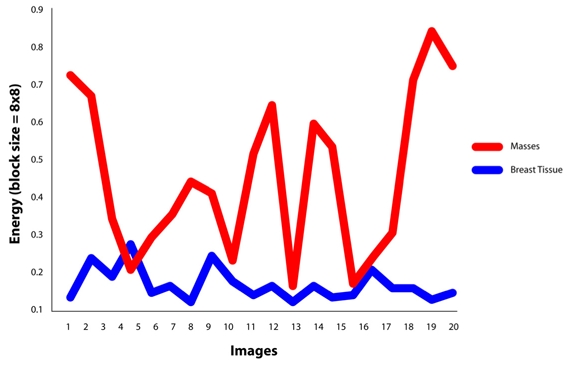
Energy value at θ=0°using tile 32x32 pixels.

**Figure 12:**
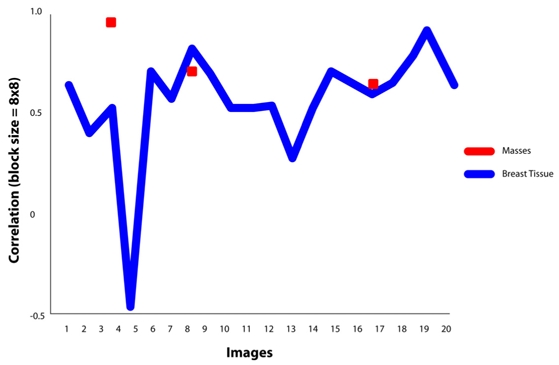
Correlation value at θ=0°using tile 32x32 pixels.

It is found that the best features for discriminating masses are the features of GLCM constructed at direction of 0°. For masses area, the contrast, homogeneity and energy ranges are 0.00-0.07, 0.96-1.00 and 0.70-1.00, respectively. Similarly for non-masses area, the contrast, homogeneity and energy ranges are 0.27-0.73, 0.63-0.80 and 0.16-0.36, respectively. It is also observed that the values of contrast, homogeneity and energy for masses area and non-masses area are highly discriminated. This has proven the usefulness of the three texture descriptors in differentiating the masses and non-masses tissues. It is also observed that, the contrast and homogeneity are two significant texture descriptors, but energy is shown to be the most effective discriminator as portrayed in Figure 8, Figure 9 and Figure 10a, respectively. Figure 10b showed that the energy extracted from tiles size of 16x16 provides less accurate than the energy of tiles sized 8x8. Note that the energy for tiles size 32x32, shown in Figure 11, failed to differentiate between masses and non-masses area. Correlation applied to any processing block has, however, shown no significance, thus we conclude that correlation cannot be used to differentiate between masses and non-masses tissues. Figure 12 portrayed the correlation result for 32x32 tiles. This result have been reported in the authors' previous work [20].

**Figure 13:**
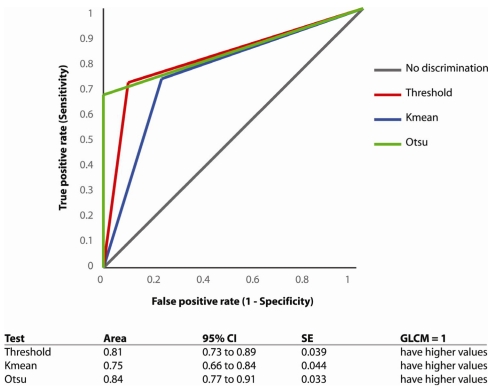
ROC curve for classification of masses using GLCM features.

Detection is done based on textural descriptors obtained from features extraction process. The sensitivity and specificity of Otsu's method in detecting masses using GLCM features is 70% and 100%, respectively. The sensitivity of local threshold is 72% and K-mean is 74% and their corresponding specificity is 88% and 74%, respectively. The overall classification accuracy is shown in Table 7. Three ROC curves computed from masses detection results of local threshold, Otsu's and K-mean methods using GLCM features are shown in Figure 13. The curve of Otsu's method with GLCM features follows the closest to the left-hand corner as compared to the others. This demonstrates that the Otsu's method is the most accurate technique in detecting masses.

**Table 7:** Sensitivity and specificity

**Segmentation method**	**Classification: GLCM Features**	
	**Sensitivity**	**Specificity**
**Threshold**	72%	88%
**K-mean**	74%	74%
**Otsu**	70%	100%

As shown in Table 8, the mean area under the ROC curve using GLCM features for Otsu’s method is 0.84. With good rating, the results also prove that Otsu’s method with GLCM features classification gives more accurate results than local threshold and K-mean in the CAD for masses detection.

**Table 8:** Area under the roc curve for masses classification using GLCM features

**Segmentation**	**Area**	**Rating**
**Threshold**	0.81	Good
**Otsu**	0.84	Good
**K-mean**	0.75	Fair

## DISCUSSION AND CONCLUSION

False positive (FP) and false negative (FN) cases are considered errors in these experiments because they will degrade the overall performance of the detection techniques. Referring to the histogram shown in Figure 13, Otsu’s method with classification based on GLCM features shows the best performance as it produces less error than the other two segmentation methods. Segmentation with Otsu’s method, with classification using GLCM features produces only 15 errors (0 FP and 15 FN) , the local threshold produces 20 errors with 6 FP and 14 FN, while K-mean performs worst with total error of 26 (13 FP and 13 FN).

**Figure 14:**
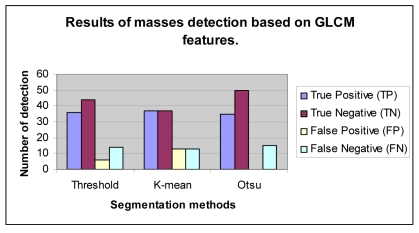
Experimental results for classification of masses using GLCM features.

In brief, Otsu’s method with GLCM features for classification of masses obtained good results in detecting any types of masses in digital mammogram. Without any complex algorithm, using a decision tree with only three GLCM features to be analysed, this detection system still able to give a good rating of area under the ROC curve with *Az* = 0.84. This approach has potential for further development because of its simplicity that will motivate online or real-time breast cancer diagnosis in providing a second opinion to radiologists.
